# Crushing Performance and Crack Development in Polymer-Fiber-Reinforced Precast Concrete Manhole Rings

**DOI:** 10.3390/ma19194061

**Published:** 2026-09-23

**Authors:** Agnieszka Głuszko, Lidia Buda-Ożóg

**Affiliations:** Department of Building Structures, Faculty of Civil and Environmental Engineering and Architecture, Rzeszow University of Technology, 35-082 Rzeszów, Poland; lida@prz.edu.pl

**Keywords:** precast concrete rings, manhole rings, polymer fibers, crushing load, post-cracking behavior, crack development, digital image correlation, ductility

## Abstract

**Highlights:**

**What are the main findings? **

• MP40 polymer fibers increased the mean ultimate crushing load by 33.6%.
• Fibers promoted distributed cracking and delayed brittle failure.
• Initial stiffness and service-load response remained essentially unchanged.

**What are the implications of the main findings? **

• The tested fiber reinforcement enhanced post-cracking load transfer.
• Distributed reinforcement enables stable stress redistribution.
• The findings support future assessments aimed at reducing conventional steel reinforcement.

**Abstract:**

Precast concrete manhole rings are exposed to localized tensile stresses and cracking during handling, transport, installation, and service. This study evaluates the effect of macro-synthetic polymer fibers on the crushing behavior, crack development, and failure mechanism of precast rings. Three reference rings and three polymer-fiber-reinforced rings made of C45/55 concrete were tested horizontally under monotonic displacement-controlled loading in accordance with EN 1917. Deformations and cracking were monitored using LVDTs, strain gauges, and digital image correlation. Both series satisfied the serviceability crack-width requirement and the minimum crushing-load criterion while showing comparable initial stiffness. The mean ultimate crushing load increased from 30.70 kN for the reference rings to 41.03 kN for the rings reinforced with MP40 macro-synthetic polymer fibers, corresponding to a 33.6% increase. At comparable advanced post-cracking deformation, the fiber-reinforced rings carried 73.6% more load. Polymer fibers promoted distributed cracking, reduced damage localization, and enabled stable load transfer after matrix cracking, whereas the reference rings developed a dominant crack and showed rapid post-peak strength reduction. The deformation criteria adopted from the fib Model Code 2010 were also satisfied. For the tested MP40 macro-synthetic fibers at a dosage of 4.0 kg/m^3^, the results demonstrate improved post-cracking resistance and ductility, supporting further assessment of reduced conventional steel reinforcement in precast manhole rings.

## 1. Introduction

Precast concrete rings are widely used as shaft units in sewer manholes and inspection chambers, where high load-bearing capacity, durability, and resistance to aggressive environmental exposure are required. According to PN-EN 1917:2004 [1] and PN-EN 476:2022-09 [2], the minimum crushing load of a ring with a diameter greater than 1 m, determined in a crushing load test, should be F_n_ = 25 kN per meter of element length. This requirement necessitates the use of solutions that ensure adequate structural stiffness and crack control.

Under ring loading, concrete rings are subjected predominantly to compression; however, cross-sectional deformation generates local tensile zones on the side opposite the applied load. Cracking initiates in these regions and significantly affects the load-bearing capacity of the element. During the production and service life of precast elements, they are subjected to various transient loading conditions that alter the distribution of internal forces. The stages of demolding, transportation, storage, and installation are particularly important because the structural system during these operations differs from the final ring action under service conditions.

During demolding, the self-weight of the precast element is carried by early-age concrete with limited strength and stiffness. Local support conditions generate bending moments and tensile stresses in zones that remain compressed during service. A similar mechanism occurs during the transportation and lifting of rings, when concentrated loads are transferred through lifting slings or support points with limited contact areas. This results in local stress concentrations and cross-sectional deformation.

Uneven support during storage or installation may additionally cause ovalization of the ring and the development of secondary circumferential stresses. In concrete elements, these effects may initiate microcracking even before the structure is placed in service. Therefore, the ability of the material to limit crack propagation and redistribute stresses after its tensile strength has been exceeded is of particular importance.

In industrial practice, the required load-bearing capacity of manhole shafts is achieved through conventional steel reinforcement, most commonly in the form of meshes placed within the joint region in concrete elements or over the full height of the ring in reinforced concrete elements. Despite its high compressive strength, concrete is characterized by limited deformability and brittle failure. Consequently, once the load-bearing capacity of the tensile zones is exceeded, cracks may propagate rapidly. The use of conventional steel reinforcement is also associated with susceptibility to corrosion in aggressive environments and increased labor requirements during precast production.

In recent years, increasing interest has been observed in the use of polymer fibers as dispersed reinforcement [3,4,5,6]. This trend is primarily associated with improved post-cracking behavior of concrete and a reduced risk of deterioration related to corrosion of conventional steel reinforcement [7]. Studies of polymer-fiber-reinforced concrete have demonstrated that fibers have only a limited effect on compressive strength [8], while significantly increasing residual strength, fracture energy, and the ability of the material to carry loads after cracking [9,10]. Fibers transfer tensile stresses by activating a crack-bridging mechanism, which limits crack propagation and delays damage development. Consequently, the loss of load-bearing capacity occurs more gradually, while the material exhibits greater ductility and resistance to brittle failure [11]. The effectiveness of macro-synthetic fibers has also been confirmed in studies of structural elements with low reinforcement ratios, such as walls [12] and tunnel lining segments [13], in which the fibers contributed to post-cracking load transfer and enabled a reduction in the amount of conventional steel reinforcement. Therefore, fibers complying with the requirements of PN-EN 14889-2:2007 [14] may constitute an alternative to conventional reinforcement in precast rings, particularly in elements exposed to transient loading conditions during demolding, transportation, storage, and installation, which generate local tensile zones and cracking.

From the perspective of precast concrete rings, the results of studies on pipe elements subjected to crushing tests are particularly relevant [15,16,17,18]. These studies demonstrated that steel fibers can provide load-bearing capacities comparable to those of conventionally reinforced elements while simultaneously increasing post-cracking load-carrying capacity and improving post-peak behavior [19]. It has also been shown that concrete pipes reinforced exclusively with polymer fibers can satisfy the normative crushing-load requirements for elements with an internal diameter of 1000 mm [20].

Despite the widespread use of precast concrete manholes in sewer systems, the load-bearing capacity and failure mechanisms of manhole rings subjected to crushing tests remain relatively poorly understood. Previous studies have focused mainly on concrete pipes, whereas the number of publications concerning manhole units manufactured in accordance with EN 1917 remains limited [10]. A previous study [10] focused on the material characterization of concrete reinforced with different types and dosages of synthetic fibers, including compressive strength, elastic modulus, flexural tensile strength, and residual strength. Based on these results, MP40 fibers at a dosage of 4.0 kg/m^3^ were selected, and a preliminary numerical assessment of the crushing behavior of precast rings was performed using the ATENA Center 2024 software (Červenka Consulting s.r.o., Prague, Czech Republic).

The next section describes the investigated materials, specimens, and full-scale crushing test procedure. Section 3 presents and discusses the load–displacement response, crack development, and deformation behavior of the tested rings, including the DIC and strain-gauge results. Finally, the main findings and directions for further research are summarized in the Conclusions section.

## 2. Materials and Methods

The experimental program included full-scale crushing strength tests of precast rings made of C45/55 concrete, designated as PFRC4 (Table 1) Three reference rings (CCR), classified by the manufacturer as concrete rings, and three rings made of concrete modified with synthetic polymer fibers (PCR) were tested. All specimens contained reinforcement made of B500C steel, consisting of a mesh made of 6 mm diameter bars, located in the upper part of the ring, as well as a single bar placed within the rebate along the diameter of the element. The same steel reinforcement arrangement was used in both CCR and PCR specimens. The reinforcement primarily served an auxiliary and technological function, contributing to crack control and maintaining the integrity of the elements during production, transportation, and installation. Nevertheless, its contribution to crack control and load redistribution after cracking cannot be neglected. Therefore, the steel reinforcement constituted a constant parameter within the experimental program, while the presence of polymer fibers was the principal variable differentiating the two series. The contribution of the steel reinforcement cannot be quantitatively separated from that of the fibers on the basis of the present test series. Accordingly, the comparison between CCR and PCR specimens should be interpreted as the incremental structural effect associated with the addition of polymer fibers to an otherwise identical steel reinforcement configuration.

**Table 1:** Concrete strength class and components of mixture PFRC4.

	Cement (kg/m^3^)	Fly Ash (kg/m^3^)	Sand/Gravel (kg/m^3^)			Water (l)	w/c	Admixture Chryso (kg/m^3^)			Fiber (kg/m^3^)
			0/2	2/8	8/16			Premia	Air LB	Xel Time	Nordica MP40
C45/55–PFRC4	380	100	730	460	470	160	0.421	3	0.4	7.6	4

Before the full-scale tests, the mechanical properties of the polymer-fiber-reinforced concrete were determined, including compressive strength, elastic modulus, and residual flexural strength, in accordance with the applicable standard procedures [21,22,23,24]. The concrete mixture used for the PCR specimens, designated as PFRC4 in the authors’ previous material-level studies, contained 4.0 kg/m^3^ of MP40 macro-synthetic polymer fibers, (Table 2). The detailed material testing program and complete results have been reported previously in [6,10]. To provide the material basis for the present structural investigation without reproducing the complete previously published datasets, the key mechanical properties of the PFRC4 concrete used to manufacture the full-scale rings are summarized in Table 3. MP40 macro-synthetic fibers at a dosage of 4.0 kg/m^3^ were selected based on the previous comparative material tests, as this mixture satisfied the residual-strength requirements specified in [25]. The crushing test was conducted with the rings positioned horizontally, in accordance with the requirements of PN-EN 1917 [1]. The tested elements had an internal diameter of 1500 mm, a wall thickness of 150 mm, and a height of 500 mm (Figure 1).

**Table 2:** Fiber properties.

	Nordica MP40	
Base Material	modified polypropylene	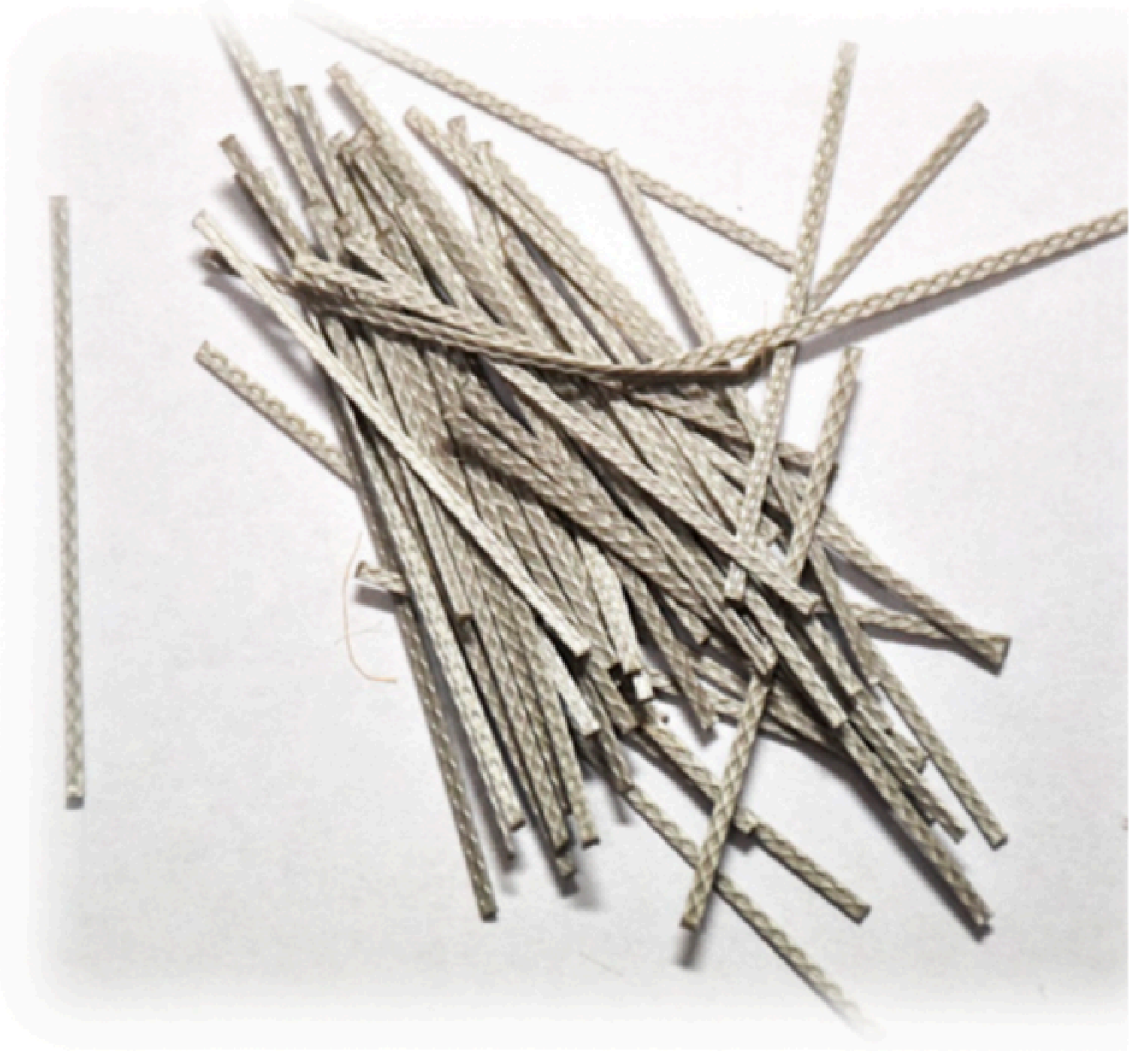
Tensile Strength (MPa)	500	
Elastic Modulus (GPa)	6	
Diameter (mm)	0.7	
Length (mm)	40	
Aspect Ratio (l/d)	57	
Form	macro monofilament	

The tested elements were positioned between steel loading plates fitted with elastomeric pads to ensure uniform load transfer and reduce local stress concentrations in the contact zones. The load was applied monotonically under displacement control until the element lost its load-bearing capacity. Due to the presence of steel reinforcement in the reference rings, the test procedure specified for reinforced-concrete rings was adopted. The same procedure was applied to the fiber-reinforced concrete rings because the test sequence is identical for both types of elements. This approach ensured consistent assessment conditions and enabled a direct comparison between the two groups. The testing procedure included two load levels specified in PN-EN 1917 [1], as illustrated in Figure 2a. First, the load was increased until the test load F_c_ was reached, corresponding to 0.67 F_n_ = 8.4 kN, where F_n_ denotes the minimum crushing load. The test load was maintained for 1 min. At this stage, the crack width was not permitted to exceed 0.3 mm over a 300 mm gauge length, and no surface damage indicative of a loss of structural integrity was allowed to occur. Subsequently, the load was increased until the ultimate crushing load F_u_ was reached, which was required to exceed F_n_. During the tests, the load–displacement response, crack development, and failure mechanism of the tested elements were recorded.

Deformations were measured using linear variable displacement transducers (LVDTs), strain gauges, and the Aramis (GOM GmbH, Braunschweig, Germany) optical measurement system based on digital image correlation (DIC) (Figure 2b). The Aramis system enabled full-field analysis of surface strains, identification of strain concentration zones, and monitoring of crack initiation and propagation throughout successive loading stages.

## 3. Results and Discussion

The load–displacement response obtained during the crushing tests revealed clear differences in the structural behavior of the reference CCR rings and the PCR rings. The displacement d presented in Figure 3 and Figure 4 represents the change in the vertical diameter of the ring along the loading axis. It was determined from the full-field DIC measurements obtained using the ARAMIS system as the relative vertical displacement between two diametrically opposite points, P0 and P180, located at the upper and lower parts of the ring cross-section, respectively. Thus, d corresponds to the reduction in the vertical diameter of the ring during loading. Figure 3 presents the individual load–displacement responses of the CCR and PCR specimens together with the corresponding mean curves. In the initial loading range, both series exhibited a similar response. The main differences became apparent after the first load maximum. The CCR specimens showed a pronounced reduction in load-carrying capacity, followed by a relatively low residual load level with increasing displacement. In contrast, the PCR specimens retained their ability to sustain load after cracking and subsequently exhibited a further increase in load with increasing displacement. The PCR curves also extended over a substantially larger displacement range, indicating a markedly greater deformation capacity.

To assess the variability of the experimental results, the standard deviation (SD) and coefficient of variation (CV) were calculated for each series. Differences between the CCR and PCR series were additionally evaluated using a two-sided Welch’s *t*-test at a significance level of α = 0.05. No statistically significant differences were found in displacement at the proof load Fc (*p* = 0.953) or at the minimum crushing load Fn (*p* = 0.924). In contrast, the difference in ultimate crushing load Fu between the CCR and PCR series was statistically significant (*p* = 0.018). However, due to the limited sample size (n = 3 per series), the statistical results should be interpreted with caution.

At the proof load F_c_ = 8.4 kN, no crack exceeding the allowable width of 0.3 mm over a 300 mm gauge length was observed in any specimen. No surface damage indicative of a loss of structural integrity was recorded.

Table 4 presents the displacements measured for the CCR and PCR rings at the proof load F_c_ and the minimum crushing load F_n_. At F_c_ = 8.4 kN, both series exhibited the same mean displacement of 0.29 mm. At F_n_ = 12.5 kN, the mean displacement was 0.37 mm for the CCR rings and 0.36 mm for the PCR rings. The negligible difference between the mean values, compared with the scatter observed within each series, confirms that the polymer fiber reinforcement did not materially alter the pre-cracking stiffness of the tested elements.

As the applied load increased, cracking was initiated in the tensile zones generated by deformation of the ring cross-section. The subsequent response of the tested elements differed markedly between the two series.

In the reference rings, once the first load peak had been reached, a rapid decrease in load-bearing capacity occurred due to the initiation of a dominant crack in the tensile zone. After the tensile strength of the concrete had been exceeded, the material lost its ability to redistribute stresses effectively, resulting in rapid damage propagation and a brittle failure mode. The force–displacement curves showed a pronounced decrease in force after the first peak and a limited capacity to sustain further loading. The mean crushing load of the CCR rings was 30.7 kN (Table 5).

A different response was observed for the PCR rings containing polymer fibers (Figure 4). After the first load peak had been reached, no sudden loss of load-bearing capacity occurred. Instead, the elements exhibited a stable post-cracking response, with the load-carrying capacity continuing to increase with increasing displacement due to fiber bridging and stress redistribution. For the tested specimens, the mean maximum load-bearing capacity of the PCR rings was 41.03 kN, which was 33.6% higher than the mean value obtained for the CCR rings (Table 5).

During the advanced post-cracking stage, the PCR elements exhibited a more uniform distribution of circumferential deformation and a gradual development of cracking. Unlike in the CCR rings, the deformations were not locally concentrated within a single dominant crack but were distributed over a larger region of the ring cross-section (Figure 5 and Figure 6). The post-cracking response of the PCR specimens should be considered as the result of the combined action of the conventional steel reinforcement and dispersed polymer fibers. The steel mesh contributes to crack control and stress redistribution in both specimen series, whereas the polymer fibers provide an additional distributed crack-bridging mechanism in the PCR rings. Because the steel reinforcement configuration was identical in both series, the observed differences between CCR and PCR can be associated with the addition of polymer fibers under the tested reinforcement arrangement. The degradation process was gradual and occurred without a sudden loss of load-bearing capacity, indicating a quasi-ductile response of the elements.

The criteria adopted in accordance with fib Model Code 2010 [9] concern the assessment of the post-cracking performance of fiber-reinforced concrete elements and the possibility of replacing conventional reinforcement with dispersed fiber reinforcement. According to this model, the use of fiber-reinforced concrete as a structural material is justified when, after crack initiation, the element exhibits adequate residual load-bearing capacity and the required ductility. The first condition (1) defines the required ability of the element to retain its load-bearing capacity in the post-cracking phase.

(1) δ_u_ ≥ 20 δ_SLS_

The parameter δ_SLS_ denotes the displacement corresponding to the serviceability limit state, whereas δ_u_ represents the ultimate displacement associated with the loss of the ability to sustain further loading. Satisfaction of this criterion indicates a high deformation capacity after the serviceability limit state has been reached. In practical terms, this means that, following crack initiation, the element maintains a stable response and remains capable of further stress redistribution owing to the crack-bridging action of the fibers. The second condition (2) concerns the ability of the element to undergo a sufficient level of deformation before reaching its maximum load-bearing capacity.

(2) δ_peak_ ≥ 5 δ_SLS_

The parameter δ_peak_ denotes the displacement corresponding to the peak load. Satisfaction of this condition indicates that, following the initiation of the first cracks, the element does not fail immediately but remains capable of sustaining increasing load with increasing deformation due to fiber activation and crack bridging. This criterion confirms the ductile nature of the structural response and the ability of the element to undergo controlled crack development.

Figure 7 compares the mean and characteristic load–displacement responses of the PCR rings and indicates the deformation parameters used to assess their post-cracking behavior. The mean curve, PCR.m, represents the average mechanical response of the tested elements, whereas the characteristic curve, PCR.k, provides a more conservative representation that accounts for the experimental scatter. Although the fib Model Code 2010 does not explicitly require the deformation criteria to be verified directly from a characteristic structural response curve, the characteristic curve was adopted in this study to provide a cautious basis for the assessment.

The characteristic load–displacement curve was adopted to provide a conservative basis for the verification, consistent with the general principle of assessing structural resistance using characteristic rather than mean response values. Accordingly, δ_SLS_, δ_peak_, and δ_u_ were determined from the characteristic PCR.k curve shown in Figure 7.

For the analyzed precast PCR rings, both conditions were satisfied:

(3) δ_u_ = 29.6 mm > 20 δ_SLS_ = 20 × 1.2 = 24 mm

(4) δ_peak_ = 13.1 mm > 5 δ_SLS_ = 5 × 1.2 = 6 mm

The fulfillment of these criteria, even on the basis of the more conservative characteristic response, confirms that the PCR rings exhibited adequate deformation capacity and maintained a stable load-bearing response after cracking.

The analysis of the ring cross-section deformation, performed on the basis of measurements obtained using the ARAMIS system, enabled a detailed assessment of the structural response of the tested rings at successive loading stages. The polar plots present the deformed shape of the ring cross-section determined from full-field displacement measurements using the digital image correlation (DIC) method implemented in the ARAMIS system. The curves labeled F0 correspond to the initial undeformed geometry of the cross-section, whereas the subsequent curves illustrate changes in the ring geometry under increasing load by tracking the vertical displacements, u_y_, and horizontal displacements, u_x_, of points distributed along the ring circumference.

To compare the structural response of the tested rings, the deformation of the cross-section was analyzed at three characteristic loading stages: within the elastic range, at the first load peak, and in the post-cracking phase. The mean force values and the corresponding displacements of selected points on the cross-section are summarized in Table 6, while the deformed shapes of the cross-section are presented in Figure 8.

Within the elastic range, both ring types exhibited a similar deformation mechanism. The cross-section retained an approximately circular shape, and the displacements of the control points remained small. The mean force at this stage was 25.47 kN for the CCR rings and 31.38 kN for the PCR rings. Despite the higher load level recorded for the PCR rings, the cross-sectional deflection was comparable to that of the reference rings. This indicates that the addition of polymer fibers did not significantly affect either the initial stiffness or the overall response of the cross-section before cracking.

At the first load peak, the deformation of the PCR rings was greater than that of the CCR rings. The first peak was reached at a mean force of 30.70 kN for the CCR rings and 37.48 kN for the PCR rings, corresponding to a 22% increase in the first-peak load for the polymer-fiber-reinforced rings relative to the reference rings. The vertical displacement of point P0 increased in magnitude from −1.74 mm for CCR to −2.19 mm for PCR, while the horizontal displacement of point P90 increased from 0.39 mm to 0.57 mm, respectively. A similar increase was observed at points P180 and P270. These results demonstrate that the PCR rings were capable of sustaining a higher load before reaching the first force maximum while maintaining a similar cross-sectional deformation mechanism.

The most pronounced differences between the responses of the CCR and PCR rings emerged after the first load peak had been reached. In the CCR rings, further deformation was accompanied by a decrease in load from 30.70 kN to 23.64 kN, indicating a progressive loss of load-bearing capacity in the post-peak phase. By contrast, the PCR rings exhibited the opposite trend, with the force increasing from 37.48 kN to 41.03 kN. Thus, after cracking, the PCR elements maintained a stable mechanical response and remained capable of sustaining additional load despite the progressive deformation of the cross-section.

At the global level, the post-cracking differences between the CCR and PCR series primarily concerned the magnitude of the sustained load rather than the overall cross-sectional deformation pattern. In both series, the same global kinematic mechanism prevailed, namely cross-sectional ovalization under the crushing load. The vertical displacement of point P0 was −14.07 mm for CCR and −14.17 mm for PCR, while the change in the horizontal dimension of the cross-section, calculated as the difference between the horizontal displacements of points P90 and P270, was 15.37 mm and 15.38 mm, respectively. Thus, a comparable level of global deformation was reached at substantially different load levels. The mean force sustained by the PCR rings was 73.6% higher than that sustained by the CCR rings, amounting to 41.03 kN and 23.64 kN, respectively. Previous studies have shown that post-cracking behavior is sensitive to fiber geometry, aspect ratio, and fiber content, as well as to the interaction between fibers and conventional reinforcement [26,27]. Therefore, the improvements observed in the present study should be regarded as specific to the MP40 fibers at a dosage of 4.0 kg/m^3^ and to the investigated ring configuration. At the local level, however, the full-field distributions of the major principal strain, ε_1_, presented in Figure 9 and Figure 10, revealed distinctly different post-cracking responses. In the CCR rings, positive principal strains were concentrated within a limited number of zones associated with dominant crack development. By contrast, the PCR rings exhibited multiple, more widely distributed zones of elevated strain along the ring circumference. This response is consistent with reduced strain localization and a more distributed cracking pattern in the presence of polymer fibers. However, the underlying fiber-bridging mechanism was inferred from the observed structural response and post-test observations rather than quantified directly through measurements of crack width, fiber pull-out, or fiber orientation.

Figure 11 presents the relationship between the force, F.TW0, and the mean strain, ε, calculated as the average of the readings from two strain gauges, TW0/1 and TW0/2, located in the upper region of the ring near the load application point. For the CCR ring, the curve covered a strain range of up to approximately 300 µm/m. The termination of the CCR curve was associated with damage to the strain gauges caused by the propagation of dominant cracks through the measurement region. The maximum recorded force was approximately 29 kN, while the measurement ended at a relatively low strain level compared with the PCR curve.

For the PCR ring, the curve covered a substantially wider strain range, extending to approximately 4300 µm/m. After a rapid increase in force to approximately 30 kN, the curve entered a region characterized by a lower slope. Within the strain range of approximately 200–1800 µm/m, the force remained mainly between 30 and 33 kN, with local fluctuations. At strains of approximately 1800–2400 µm/m, further local changes in the curve were observed. Subsequently, the force stabilized at approximately 35 kN and remained close to this level until the end of the measurement.

The post-test observations additionally confirmed the crack-bridging action of the polymer fibers. As shown in Figure 12, the fibers remained engaged across the opened crack, contributing to residual tensile stress transfer after loss of continuity of the concrete matrix.

## 4. Conclusions

Based on the full-scale crushing tests performed on the precast concrete rings, the following conclusions can be drawn:

• Both the reference CCR rings and the PCR rings satisfied the standard requirements for the minimum crushing load. For the tested elements with a height of 0.5 m, the required value was F_n_ = 12.5 kN. The mean ultimate crushing loads were 30.70 kN for the CCR rings and 41.03 kN for the PCR rings. Thus, the PCR rings containing MP40 fibers at 4.0 kg/m^3^ achieved a 33.6% higher mean load-bearing capacity than the reference elements. Within the service-load range, including the proof load Fc and the minimum crushing load F_n_, both ring types exhibited comparable initial stiffness. The mean displacements at Fc were identical for both series, while the differences at F_n_ were negligible. This indicates that the addition of polymer fibers did not significantly alter the pre-cracking response of the elements.
• The principal differences between the CCR and PCR rings became evident after cracking. In the CCR rings, reaching the first load peak was followed by a reduction in load-carrying capacity associated with damage localization within a dominant crack. By contrast, the PCR rings maintained a stable mechanical response and remained capable of sustaining further loading as deformation increased.
• The cross-sectional deformation analysis showed that the polymer fibers did not fundamentally alter the deformation mode of the rings, which in both series was governed by ovalization of the cross-section. The key difference concerned the load sustained at a comparable level of deformation. In the post-cracking phase, the mean force carried by the PCR rings was 73.6% higher than that carried by the CCR rings, confirming the greater effectiveness of the cracked cross-section when polymer fibers were present.
• The PCR rings exhibited distributed crack development and a less abrupt degradation response than the CCR rings. This behavior is consistent with the crack-bridging action of the fibers, which may have contributed to reduced damage localization and improved stress redistribution after cracking (Figure 12).
• Satisfaction of the adopted deformation criteria according to the fib Model Code 2010 [9] confirmed the ability of the PCR rings to maintain a stable post-cracking response. The obtained values of δ_u_ and δ_peak_ indicate that the polymer-fiber-reinforced elements exhibited adequate ductility and the ability to undergo further deformation while maintaining a high load level.

The test results demonstrate that, for the investigated ring configuration, MP40 macro-synthetic fibers at a dosage of 4.0 kg/m^3^ improved the post-cracking performance by increasing load-bearing capacity, reducing the brittle nature of failure, and enhancing the ability of the elements to redistribute stresses in a controlled manner. These findings are specific to the fiber type, dosage, and ring configuration investigated in this study and should not be directly generalized to other fiber-reinforcement systems. The results provide a basis for further assessment of the potential to reduce the amount of conventional steel reinforcement used in this type of precast element. Future research will focus on full-scale rings with reduced steel reinforcement, including its partial and potentially complete replacement by dispersed polymer fiber reinforcement, with particular attention to load-bearing capacity, crack development, deformation response, and failure mechanism.

## Figures and Tables

**Figure 1:**
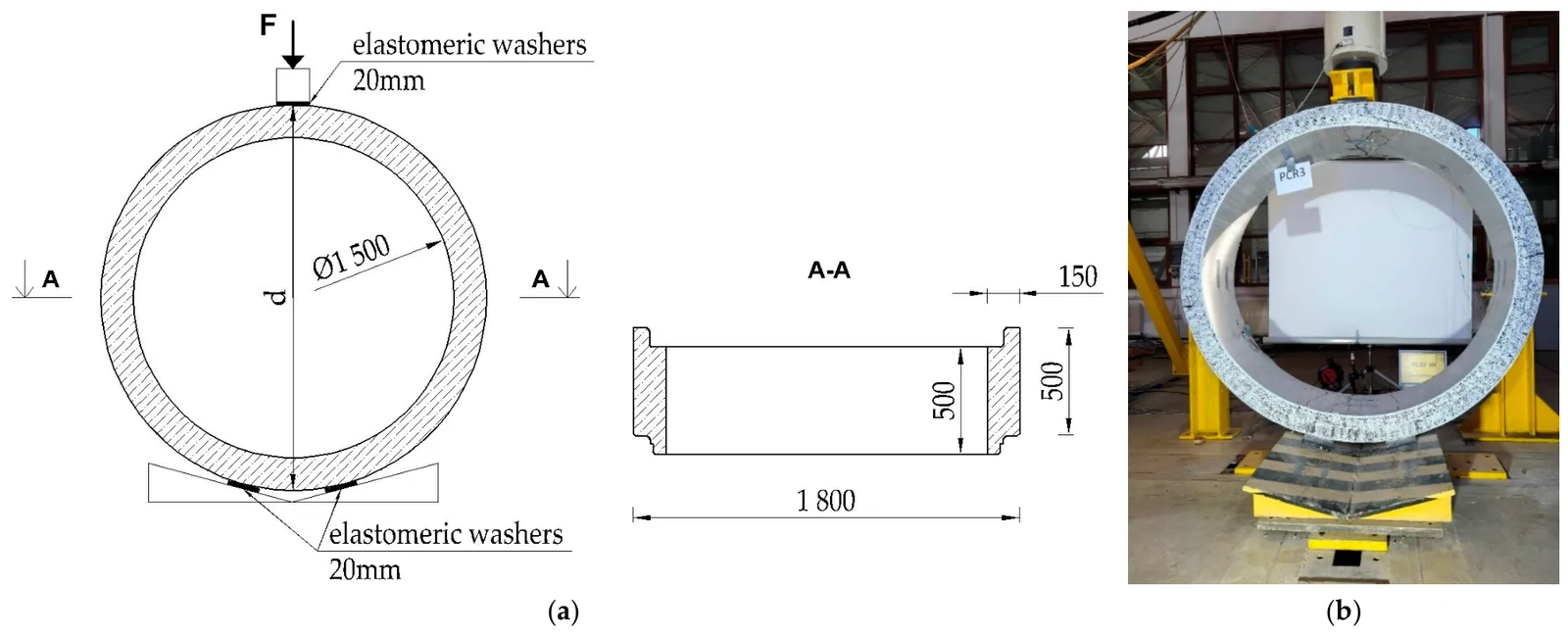
Experimental setup and geometry of the tested prefabricated concrete rings subjected to crushing load: (**a**) geometry and loading scheme, where F denotes the applied crushing load; (**b**) experimental setup.

**Figure 2:**
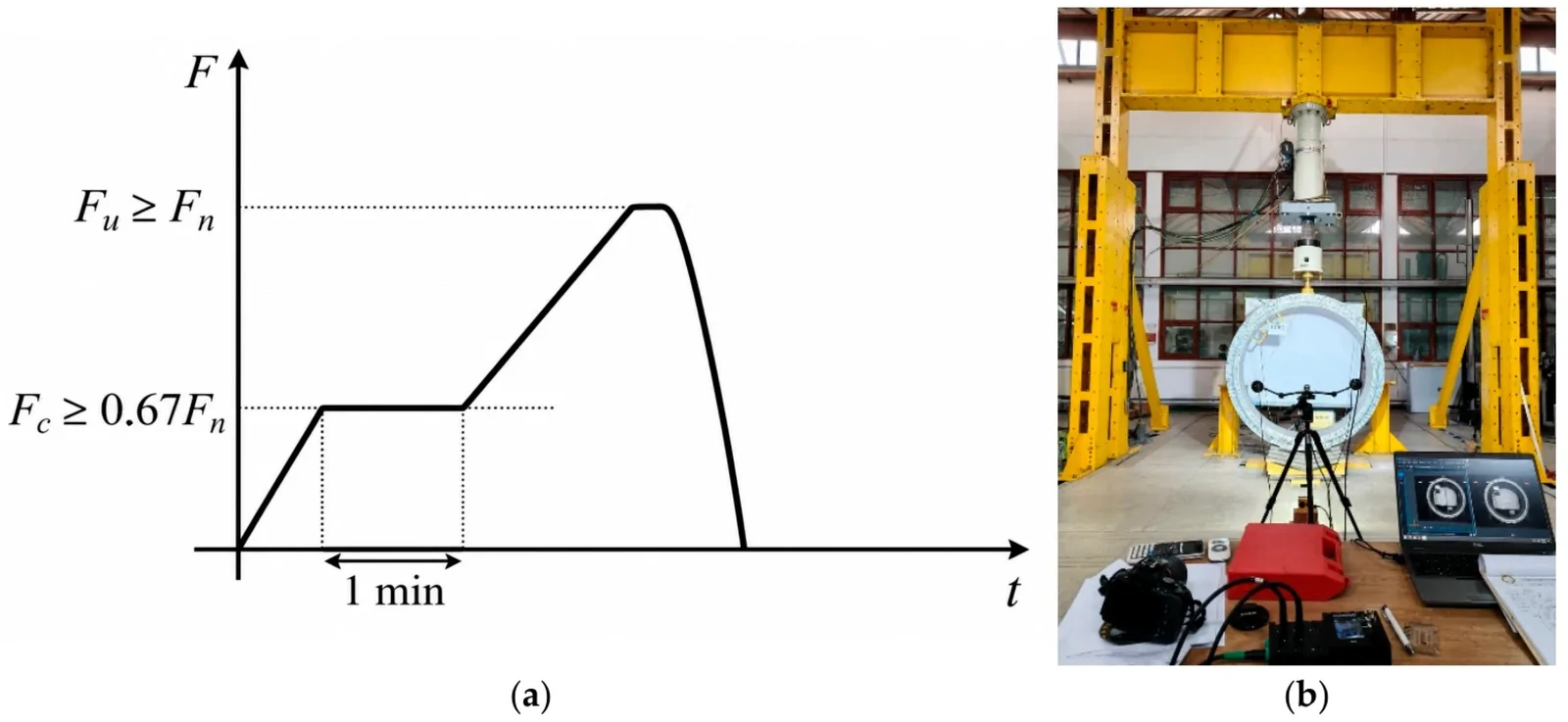
Test procedure and experimental setup for the crushing test of concrete rings: (**a**) prescribed load pattern; (**b**) full-scale experimental setup.

**Figure 3:**
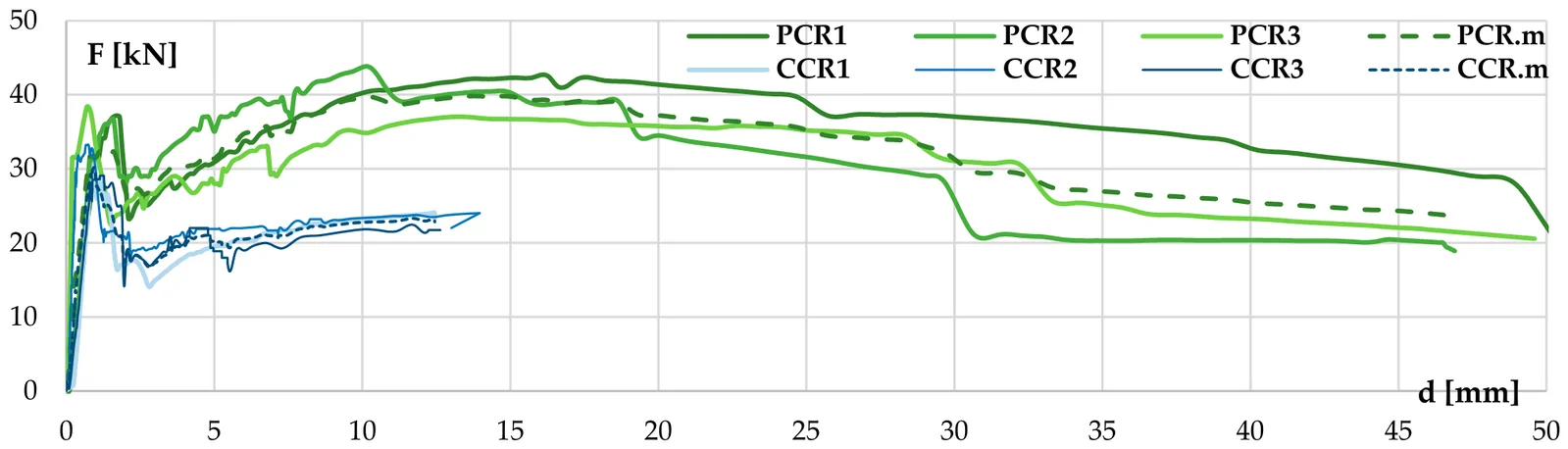
Load–displacement curves of CCR and PCR rings under crushing load.

**Figure 4:**
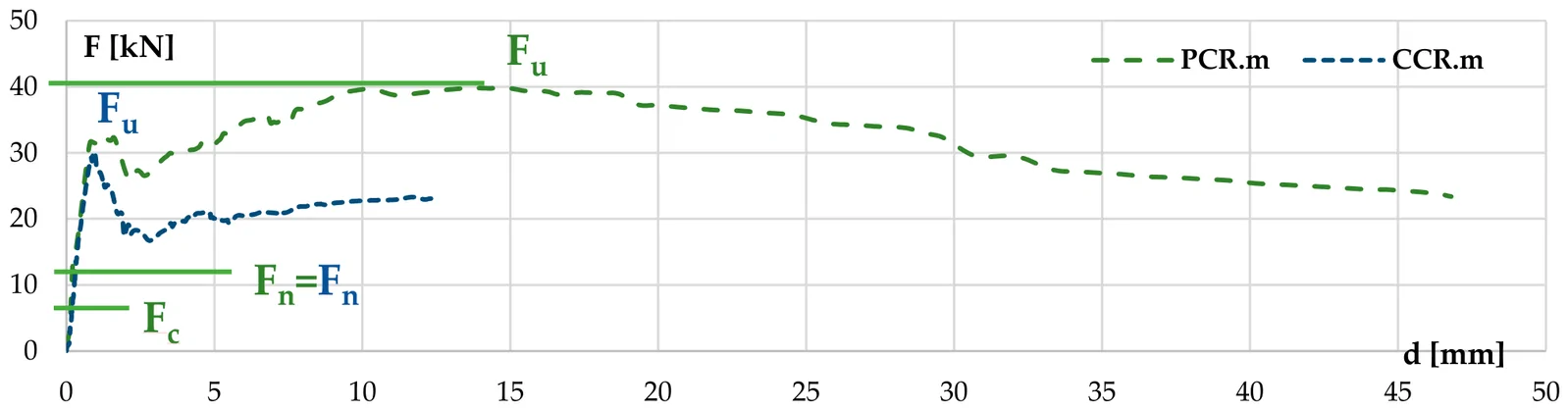
Mean load–displacement curves of CCR and PCR rings under crushing load.

**Figure 5:**
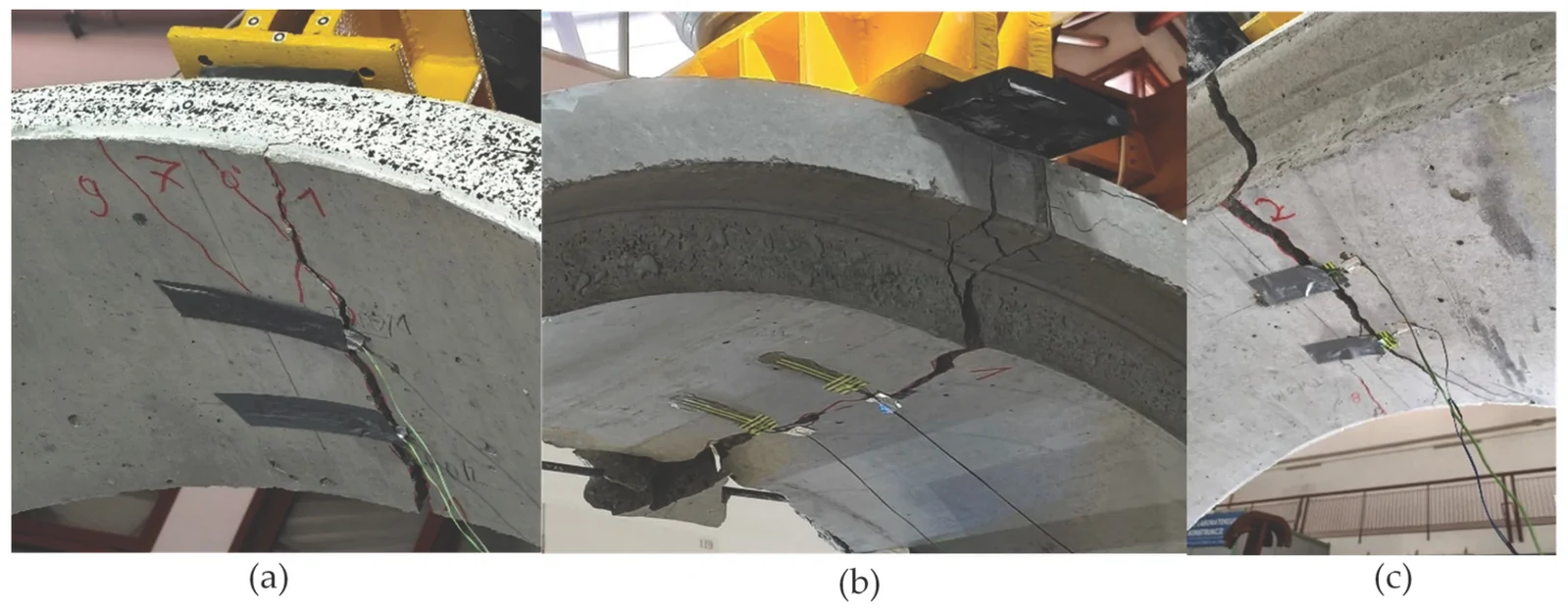
Dominant single crack observed in the reference rings: (**a**) CCR1; (**b**) CCR2; (**c**) CCR3.

**Figure 6:**
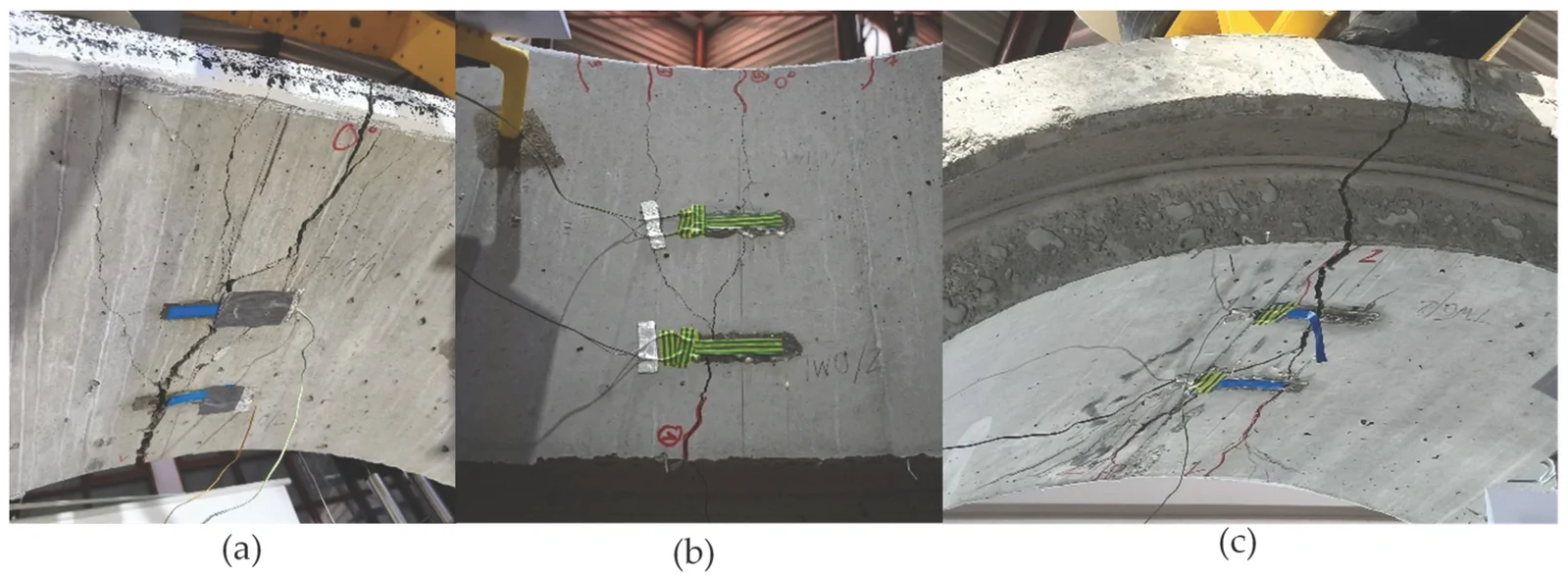
Distributed cracking pattern observed in PCR rings: (**a**) PCR1; (**b**) PCR2; (**c**) PCR3.

**Figure 7:**
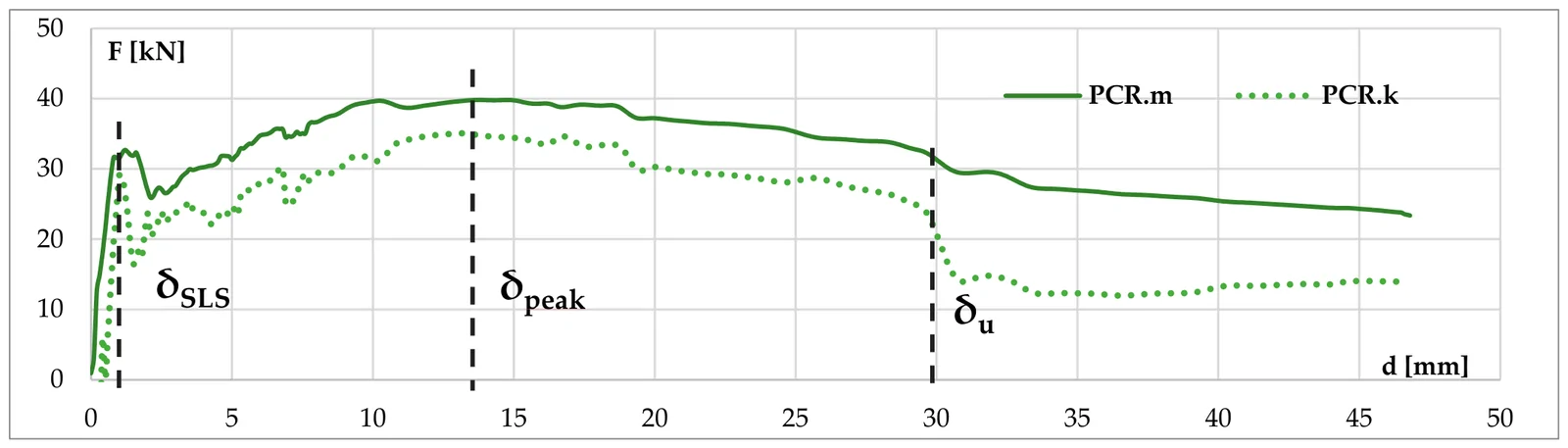
Load–displacement curves of PCR rings based on mean and characteristic values.

**Figure 8:**
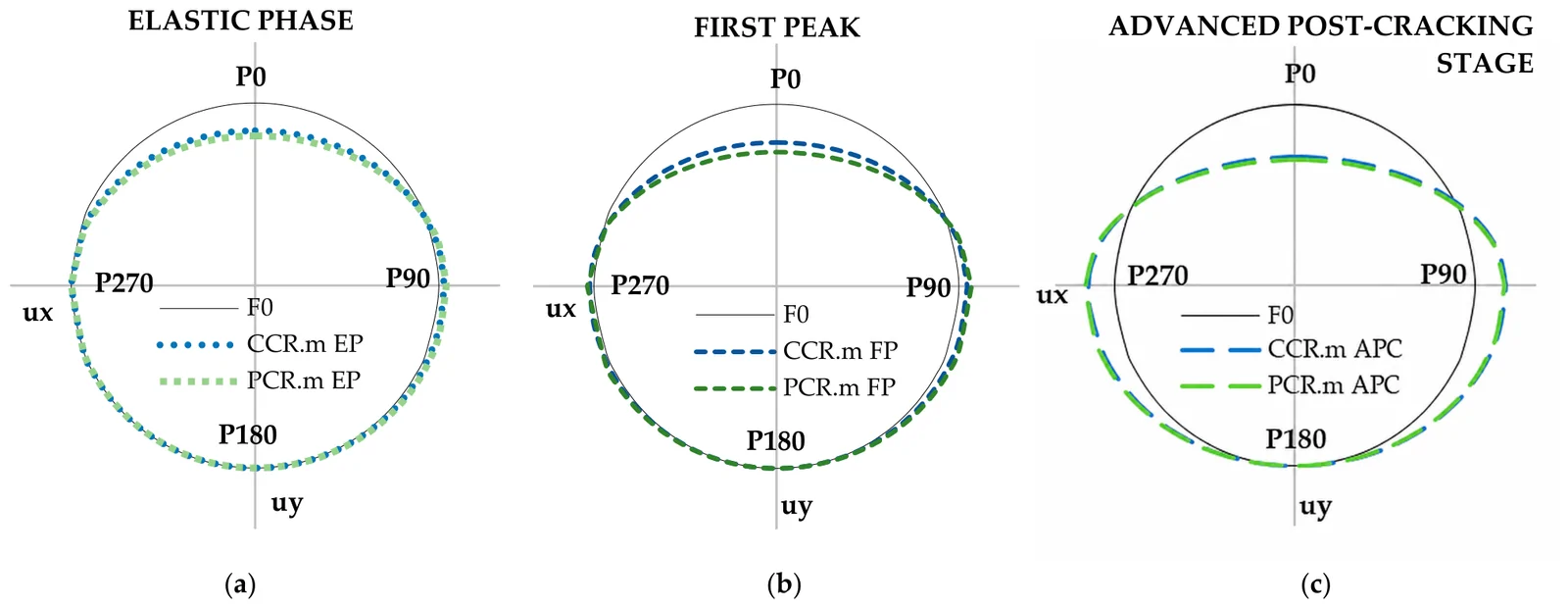
Comparison of mean cross-sectional deformation patterns of CCR and PCR rings at selected characteristic load stages: (**a**) elastic phase (EP), (**b**) first peak (FP), and (**c**) advanced post-cracking stage (APC).

**Figure 9:**
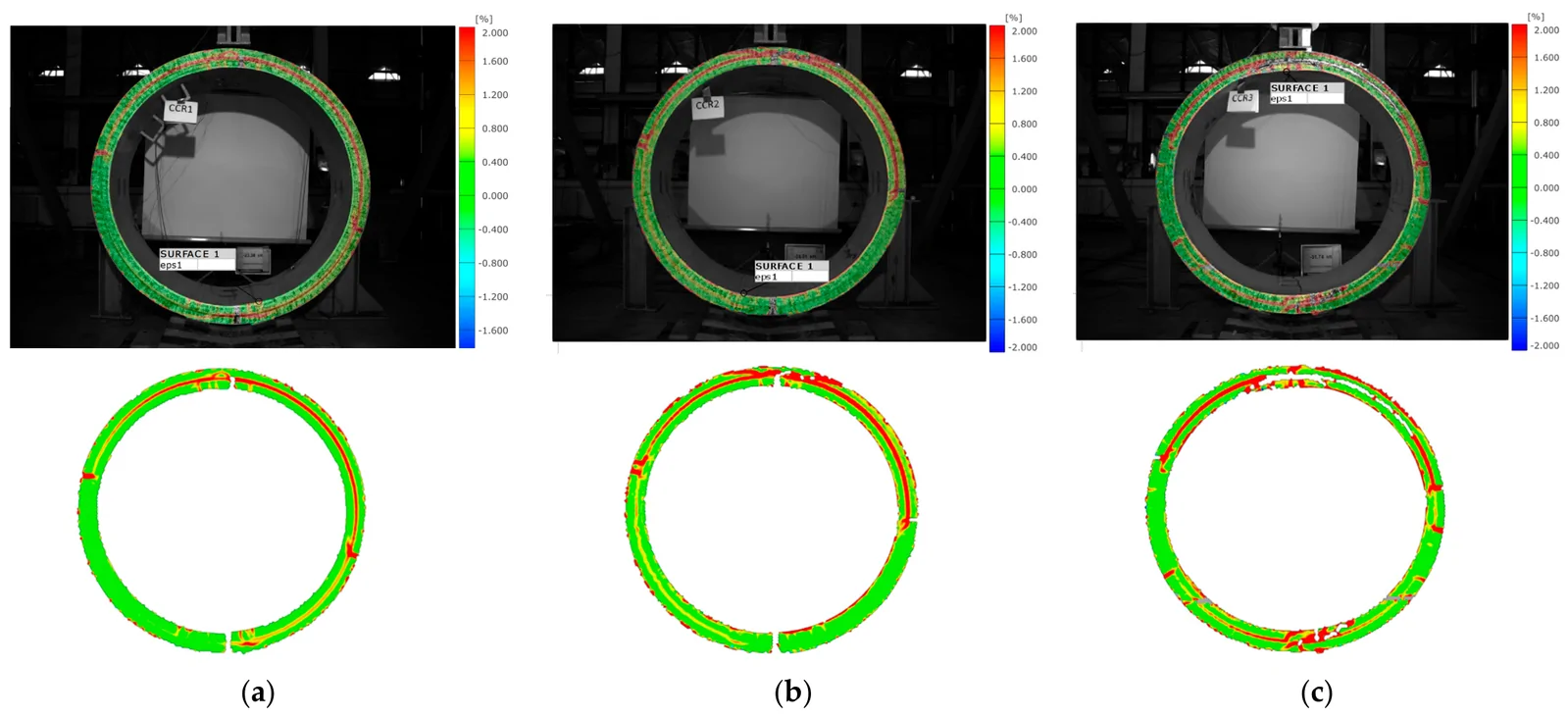
Full-field distributions of the major principal strain, ε_1_, obtained using the DIC method for the reference concrete rings at the analyzed post-cracking stage: (**a**) CCR1, (**b**) CCR2, and (**c**) CCR3. Upper row: strain fields superimposed on the specimen images; lower row: isolated strain maps.

**Figure 10:**
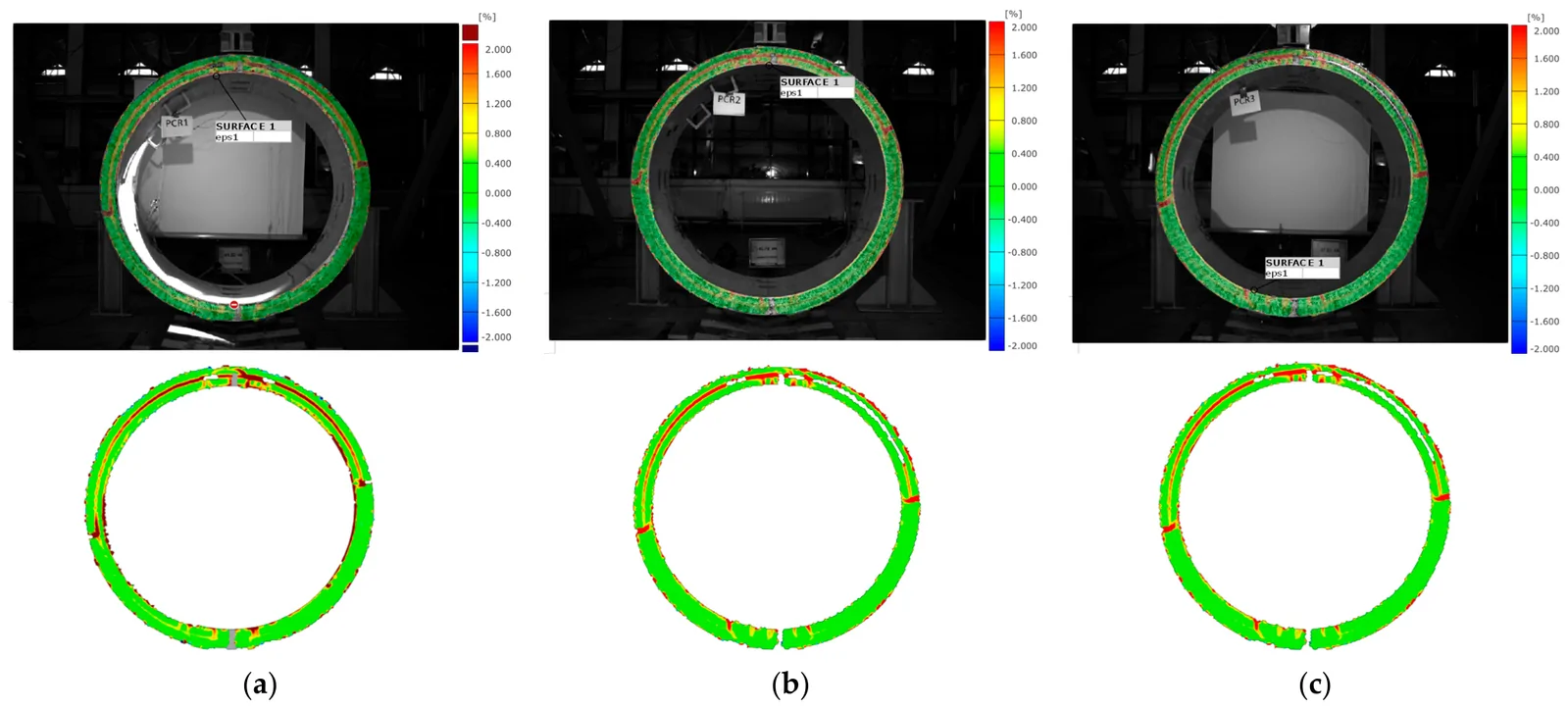
Full-field distributions of the major principal strain, ε_1_, obtained using the DIC method for the PCR concrete rings at the analyzed post-cracking stage: (**a**) PCR1, (**b**) PCR2, and (**c**) PCR3. Upper row: strain fields superimposed on the specimen images; lower row: isolated strain maps.

**Figure 11:**
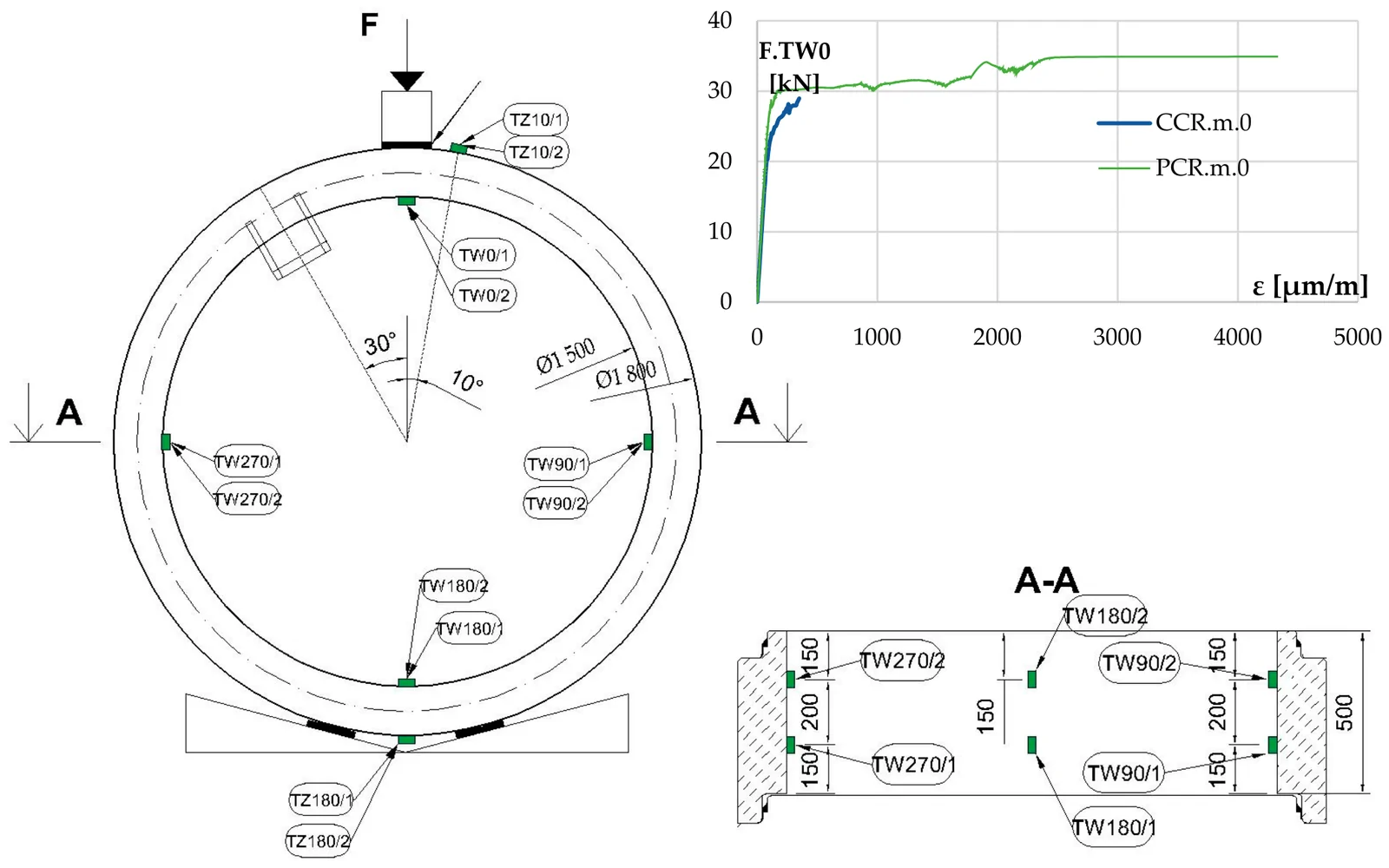
Force–strain relationship based on mean strain values from gauges located in the upper zone of CCR and PCR rings.

**Figure 12:**
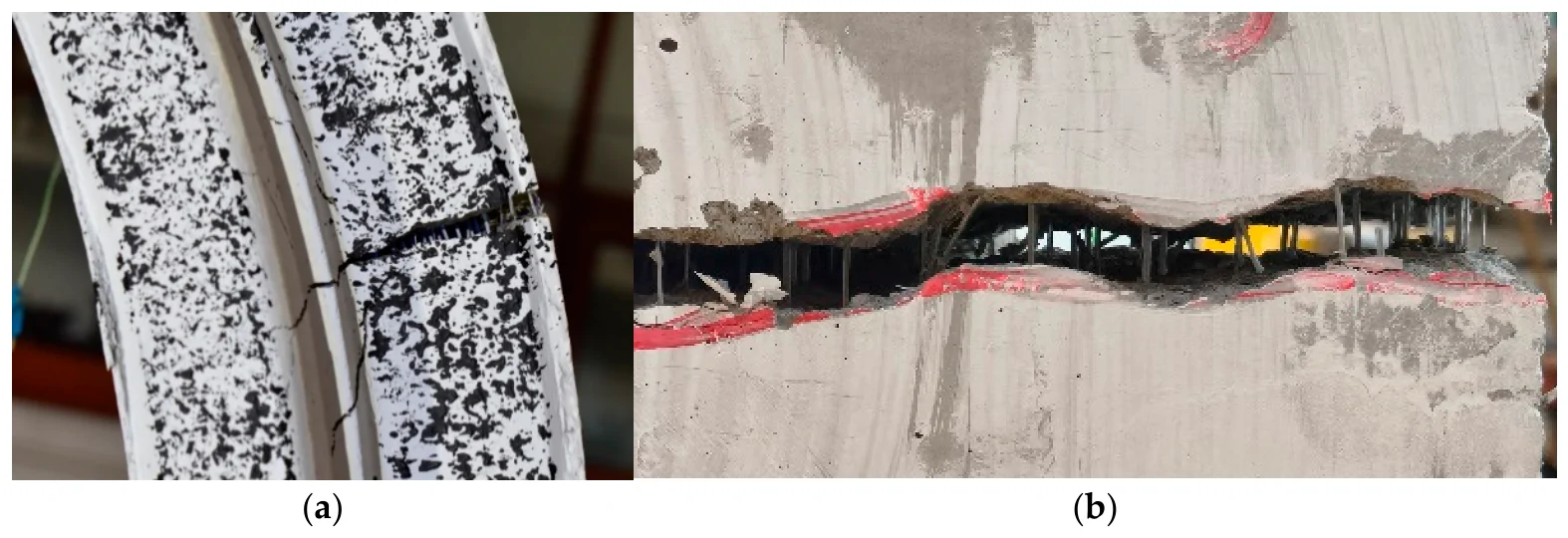
Representative crack in a PCR ring after the crushing test: (**a**) crack opening through the ring wall; (**b**) polymer fibers bridging the crack.

**Table 3:** Selected mechanical properties of PFRC4 concrete used in the full-scale rings.

Property	Mean Value	SD	n
f_c,cube_ [MPa]	86.8	0.8	6
f_c,cyl_ [Mpa]	85.5	0.8	4
E_cm_ [Gpa]	44.8	1.5	3
f_R1_ [Mpa], CMOD = 0.5 mm	1.77	0.12	3
f_R2_ [Mpa], CMOD = 1.5 mm	2.53	0.23	3
f_R3_ [Mpa], CMOD = 2.5 mm	2.83	0.32	3
f_R4_ [Mpa], CMOD = 3.5 mm	2.57	0.21	3

**Table 4:** Displacements of CCR and PCR rings at the proof load F_c_ and the minimum crushing load F_n_.

	Displacement d [mm]	
Sample	F_c_ = 8.4 kN	F_n_ = 12.5 kN
CCR1	0.42	0.49
CCR2	0.12	0.17
CCR3	0.32	0.45
CCR.m	0.29	0.37
SD	0.15	0.17
CV [%]	53.29	47.12
PCR1	0.34	0.46
PCR2	0.33	0.42
PCR3	0.17	0.19
PCR.m	0.28	0.36
SD	0.10	0.15
CV [%]	34.07	40.86

**Table 5:** Ultimate crushing loads of precast concrete manhole rings.

Sample	Displacement d [mm]	Fu [kN]
CCR1	0.98	29.76
CCR2	0.73	33.27
CCR3	1.18	29.07
CCR.m	0.96	30.70
SD	0.23	2.25
CV [%]	23.40	7.34
PCR1	15.77	42.36
PCR2	10.79	43.70
PCR3	13.34	37.03
PCR.m	13.30	41.03
SD	2.49	3.53
CV [%]	18.72	8.60

**Table 6:** Mean load and corresponding horizontal and vertical displacements of selected cross-sectional points in CCR and PCR rings at selected characteristic load stages.

Ring Type	Stage		P0	P180	P90	P270
		F_m_ [kN]	u_y_ [mm]		u_x_ [mm]	
	EP	25.47	−1.24	−0.65	0.24	0.00
CCR.m	FP	30.70	−1.74	−0.79	0.39	−0.19
	APC	23.64	−14.07	0.14	8.19	−7.18
			P0	P180	P90	P270
		F_m_ [kN]	u_y_ [mm]		u_x_ [mm]	
	EP	31.38	−1.53	−0.78	0.33	0.00
PCR.m	FP	37.48	−2.19	−0.91	0.57	−0.30
	APC	41.03	−14.17	−0.27	7.57	−7.81

Note: EP—elastic phase; FP—first peak; APC—advanced post-cracking stage. F_m_ denotes the mean load corresponding to the analyzed stage. Negative values indicate displacement in the negative direction of the corresponding coordinate axis shown in Figure 8.

## Data Availability

The original contributions presented in this study are included in the article. Further inquiries can be directed to the corresponding author.
